# Primary leiomyosarcoma of the middle ureter: A rare case report with literature review

**DOI:** 10.1002/ccr3.5409

**Published:** 2022-02-23

**Authors:** Hamdy A. Aboutaleb, Praphool Khurana, Yassin M. El‐Shahat

**Affiliations:** ^1^ Urologist Burjeel Hospital Abu Dhabi UAE

**Keywords:** immunohistochemistry, leiomyosarcoma, tumor, ureter

## Abstract

Leiomyosarcoma is a rarely seen neoplasm of the ureter. Malignant tumors of smooth muscle of the ureter are extremely rare, and about 22 cases of leiomyosarcoma of ureter have been reported to date. A 57‐year‐old diabetic Pakistani man presented with a dull ache pain in the right flank. Past surgical history was three ureteroscopic surgeries for a ureteric stricture. Computed tomography showed a stricture with a peri‐ureteral soft tissue mass of 11 mm x 5 mm at the middle third of the ureter at the level of common iliac vessels. laparoscopic excision with safety margin and right ureterovesical reimplantation is performed. Diagnosis of leiomyosarcoma of the right ureter was made, and one iliac lymph node was excised and was positive for tumor by pathologic examination. Although leiomyosarcoma is rarely seen in urinary tract, it should be considered in the differential diagnosis of ureteral stricture disease and retroperitoneal tumors.

## Graphical abstract

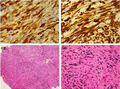

## 1 INTRODUCTION

Transitional cell carcinoma is the commonest malignancy in urinary tract and was found in more than 90% of upper urinary tract tumors. In non‐transitional cell carcinoma, the most common is squamous cell carcinoma (0.7 to 7%) and adenocarcinoma (1%). Multiple types of sarcomas have also been reported in the upper urinary tract, including leiomyosarcoma, plasmacytomas, and angiosarcomas. The sarcomas are rapidly growing tumors, invade the adjacent structures. Leiomyosarcomas have early metastasis to mesentery, lungs, liver, and regional lymph nodes.^1^, ^2^, ^3^, ^4^

Hematuria is absent in most cases due to non‐involvement of ureteral mucosa..^5^, ^6^ In two‐third cases involves distal ureter resulting in a ureteric obstruction. Synchronous bladder involvement was found in 40% of cases.^4^ Leiomyosarcoma is a rare neoplasm of the ureter with less number of reports in the literature. We report the clinical features, histology, imaging, and treatment of ureteral leiomyosarcoma in a 57‐year‐old male Pakistani patient.^7^, ^8^, ^9^, ^10^

### 1.1 Case report

A 57‐year‐old Pakistani man presented to our hospital with a dull pain in the right flank, which had persisted for 4 years. He complains of right flank pain and clinically diagnosed as ureteric stricture. The patient is diabetic but controlled by medical treatment. Past surgical history of the patient was three ureteroscopic surgeries and double J stenting three times for the stricture and one laser cut for the same stricture with stenting for 6 weeks in different institutions in the last few years. Past history of appendectomy when he was a twenty‐year‐old with ugly scar in the right iliac fossa was seen. The scar was due to complicated appendectomy wound with incisional hernia and repaired with mesh. The patient has unexplained polyuria, and he passes 5 liters of urine every day without consultation. Our team work formed of urologist, nephrologist, endocrinologist, and oncologist.

### 1.2 Laboratory investigation

Urine examination identified microscopic hematuria; Blood sugar is normal, renal function tests are within normal. Urine PH is normal and repeated many times with normal results. Anti‐diuretic hormone is evaluated with normal results. Potassium results are borderline results and when repeated, postoperatively, he had hypokalemia associated with ileus in the postoperative course.

### 1.3 Radiological findings

**FIGURE 1:**
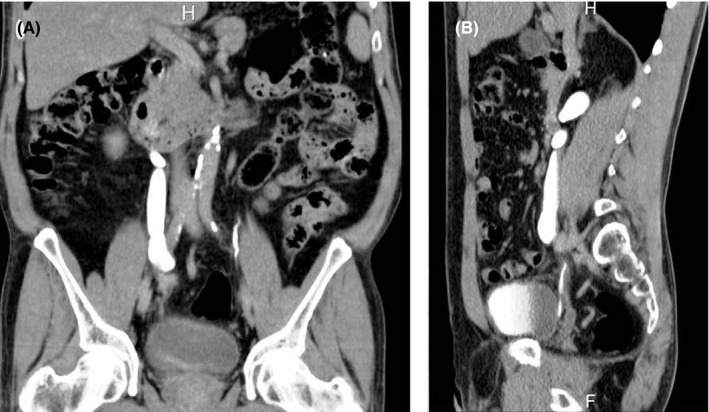
(A, B). Computed tomography (CT‐KUB) (Figure 1) showed the presence of a stricture of 11 mm x 5 mm at the middle third of the ureter at the level of common iliac vessels with dilation of the proximal ureter and renal pelvis. The lumen of the affected ureter appeared to be irregular in shape

Ultrasonography identified right moderate hydronephrosis with dilation of the proximal ureter. Computed tomography abdomen and pelvis (CT‐KUB) (Figure 1A, B) showed the presence of a stricture of 11 mm x 5 mm at the middle third of the ureter at the level of common iliac vessels with dilation of the proximal ureter and renal pelvis. The peri‐ureteral enhanced soft tissue mass was seen 11 mm x 5 mm as reported by CT‐KUB with contrast. Furthermore, the lumen of the affected ureter appeared to be irregular in shape. After injection of contrast medium, the mass was greatly enhanced, with a clear portion in the nearby tissues. The distance of the mass from the renal pelvis was about 15 cm.

### 1.4 Procedure

Diagnostic ureteroscopy revealed a narrowing of the lumen of the ureter without any mass seen in the ureteric lumen. Under general anesthesia, diagnostic laparoscopy revealed the ureteric stricture at the area of crossing iliac vessels, and proximal ureter is dilated. The ureteric stricture area is resected with safety margin 1 cm above. The proximal tortuous ureter is straightened and re‐implanted in submucosal tunnel in the bladder with Boari flap using 4/0 Vicryl. A double J stent 5 Fr/ 24 cm is fixed for 6 weeks. Postoperatively, the patient had distended abdomen with urine output exceeding 7 liters every day associated with hypokalemia. Potassium is corrected, and bowel movements are resumed. The patient is investigated for diabetes insipidus, and nephrogenic type is diagnosed. Diabetes mellitus is controlled well in the postoperative course. The patient is discharged after 10 days of the surgery with oral potassium and medicines of diabetes mellitus.

### 1.5 Pathologic examination

**FIGURE 2:**
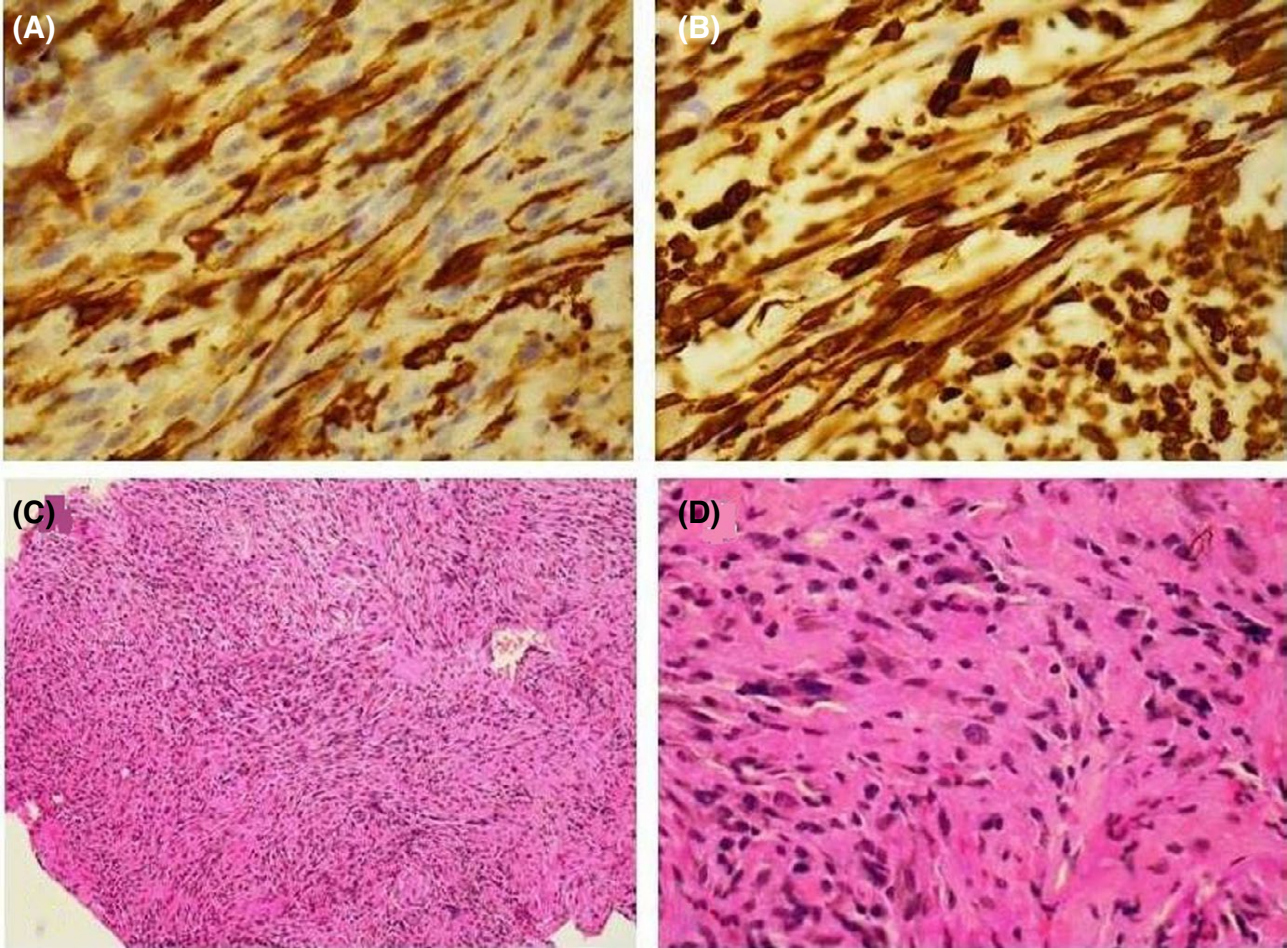
(A). Immunohistochemical staining and microscopic study showing periureteric mass positive for smooth muscle actin (40 X, IHC). (B) was positive for vimentin confirmed the diagnosis of Leiomyosarcoma. (C) Interlacing bundles of pleomorphic spindle cells with storiform pattern in periureteric mass (10x, H & E) (1). (D) Periureteric mass showing elongated, pleomorphic spindle cells (40 x, H & E)

Pathologic examination showed that leiomyosarcoma of the ureter. The cancer was composed of fascicles of interlacing, moderately large, spindle‐shaped cells, with abundant eosinophilic cytoplasm. Immunohistochemistry showed strong staining for smooth muscle actin (SMA) (Figure 2A‐D). A diagnosis of leiomyosarcoma of the right ureter was made, and one iliac lymph node was excised and was positive for tumor by pathological examination. The cutting margin of the ureter near the bladder was negative, and the kidney was intact. Adjuvant chemotherapy was suggested after surgery. The patient's convalescence was uneventful at 6 months after surgery.

### 1.6 Follow‐up

**FIGURE 3:**
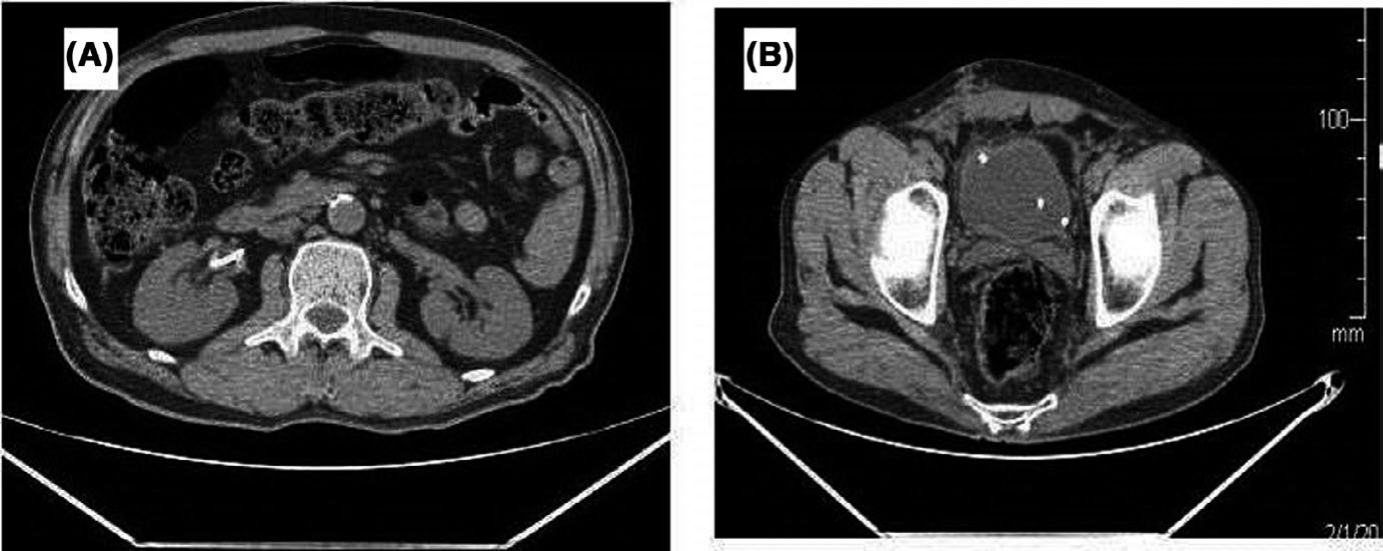
(A, B) Postoperative CT‐KUB revealed the double J in situ and high level of urinary bladder due to Psoas hitch

PET CT scan is performed after diagnosis of leiomyosarcoma to find out any metastasis, but the result was negative. The patient did not have recurrence at 3 and 6 months CT scan examination (Figure 3A, B). We recommended adjuvant radiotherapy or chemotherapy of tumor bed to our patient hoping for a better prognosis though radio‐sensitivity of leiomyosarcoma is doubtful.

## 2 DISCUSSION

Leiomyosarcoma is a rare soft‐tissue tumor that accounts for 10–20% of soft‐tissue sarcoma, is generally seen in middle‐aged patients, and afflicts women more frequently than men.^11^ Its usual locations are retroperitoneal, intra‐abdominal, cutaneous, and subcutaneous. Leiomyosarcoma is a highly malignant tumor with an extremely poor prognosis. The 5‐year overall survival rate in patients with soft‐tissue sarcoma of all stages remains poor, at only 50–60%, and 5‐year disease‐free survival is rare.^7^, ^8^, ^10^, ^11^, ^12^, ^13^, ^14^, ^15^ To our knowledge, a literature review has revealed only less than 22 cases of primary leiomyosarcoma of the ureter. We did PubMed search for all leiomyosarcoma cases in urogenital system. Sixty‐one cases are reported in humans in the last 46 years. From 1975 to 2021, they reported 22 cases in the ureter and 21 cases in the urinary bladder. Leiomyosarcoma was reported in kidney in 31 cases. Moreover, three cases were reported in the prostate and two cases in the urethra (Table 1).

**TABLE 1:** PubMed search for previous publications for primary leiomyosarcoma of ureter in humans

	DATE OF PUBLICATION	TITLE	JOURNAL	MAIN AUTHOR
1	1975	Primary sarcoma of the ureter. Study of literature apropos of a personal case	J Urol Nephrol (Paris)	Giraud B.et al
2	1979	Primary leiomyosarcoma of the ureter in a dog.	J Am Vet Med Assoc	J L Berzon et al
3	1980	Primary leiomyosarcoma of ureter.	Urology	Roemer CE et al
4	1981	Primary neoplasms of the ureter.	J. Urol	Werth DD et al
5	1983	Primary leiomyosarcoma of the ureter: a case report with electron microscopy.	J Urol.	Rushton HG et al
6	1984	Primary ureteral leiomyosarcoma	Scand J Urol Nephrol	Gislason T et al
7	1987	Primary leiomyosarcoma of the ureter	Actas Urol Esp.	Chesa PN et al
8	1988	Leiomyosarcoma of the ureter	Eur Urol.	Madgar I et al
9	1993	Non‐urothelial tumors of the urinary tract	Verh Dtsch Ges Pathol	Mikuz G. et al
10	1994	Primary leiomyosarcoma of the ureter	Urol Int	Nakajima F et al
11	1996	Primary leiomyosarcoma of the ureter.	J Surg Oncol	Griffin JH et al
12	2003	Primary ureteral leiomyosarcoma: a rare cause of obstructive uropathy	Arch Esp Urol.	Márquez‐Moreno AJ et al
13	2006	A case of primary leiomyosarcoma of the ureter	Hinyokika Kiyo	Shirotake S et al
14	2007	Poorly differentiated transitional cell carcinoma versus leiomyosarcoma of the ureter: different defects in tumor suppressor genes.	Histopathology	Tzen CY et al
15	2008	Primary leiomyosarcoma of the ureter.	Asian J Surg.	Chen Lv _et al_
16	2011	Prognostic factors and clinical outcomes of urological soft tissue sarcomas.	Korean J Urol.	Lee G.
17	2012	Leiomyosarcoma of the ureter: a rare case	Diagn Interv Imaging	Aubert E et al
18	2014	Carcinosarcoma of the Ureter with a Small Cell Component: Report of a Rare Pathologic Entity and Potential for Diagnostic Error on Biopsy	Case Rep Pathol	Newsom K. et al
19	2014	Retroperitoneal Sarcoma Involving Unilateral Double Ureter: Management, Treatment and Psychological Implications	Case Rep Oncol	Leanza V. et al
20	2016	Bladder chondrosarcoma plus urothelial carcinoma in recurred transitional cell carcinoma of the upper urinary tract: a case report and literature review	World J Surg Oncol.	Cho M.H. et al
21	2019	Primary carcinosarcoma of the ureteropelvic junction associated with ureteral duplication: A case report	Medicine (Baltimore	Tsuji K. et al
22	2021	Sarcomatoid urothelial carcinoma of the ureter with heterologous elements of chondrosarcoma and osteosarcoma, and concurrent divergent squamous differentiation: A rare case report	Urol Case Rep.	Attia A. et al

In our case report, the metastasis to iliac nodes is positive leiomyosarcoma at short‐term follow‐up for 6 months after surgical excision is good.

An important aspect of the management of leiomyosarcoma is to differentiate the lesion from rhabdomyosarcoma and other spindle‐cell neoplasms. This is especially difficult with poorly differentiated high‐grade lesions. On immunohistochemistry, leiomyosarcoma is negative for epithelial markers (cytokeratins and epithelial membrane antigens). Positivity for desmin and SMA shows the smooth muscle origin of the tumor and gives a definitive diagnosis. Negative myoglobin, cytokeratin, and S‐100 help to rule out rhabdomyosarcoma, sarcomatoid carcinoma, and melanoma, respectively.^16^, ^17^ Leiomyosarcoma shows nodular aggregates and densely packed, interlacing bundles of smooth muscle cells in the center. At the margin, strands of smooth muscle cells extend between collagen bundles. Areas of lesser differentiation are present in all tumors to varying degrees, including irregularly shaped, anaplastic nuclei, and atypical giant cells with bizarre nuclei. A high mitotic rate remains the main criterion for leiomyosarcoma, and other histopathological features of the tumor include atypical smooth muscle cells with peripheral extension into the surrounding tissue, pleomorphism, giant cells, and necrosis of the tumor and peripheral tissue. Only some rhabdomyosarcomas contain cells that are recognizable as rhabdomyoblasts, by virtue of their cytoplasm exhibiting cross‐striation, which is best revealed by phosphotungstic acid‐hematoxylin staining. Round cells of the embryonal variant tend to have cytoplasmic rims that are stained red by trichrome. The botryoid type is a variant of the embryonal type and is found mainly in mucosa‐lined hollow organs. Cells with elongated eosinophilic cytoplasm may be suggestive but not necessarily diagnostic of rhabdomyosarcoma and may be found in the pleomorphic type. A sclerosing variant has cords of small, round malignant cells embedded in a densely hyalinized matrix that has a chondroid and osteoid appearance. Immunohistochemical staining of deparaffinized sections shows expression of desmin‐ and muscle‐specific actin in the great majority of leiomyosarcomas. Anti‐desmin staining is found in 47–85% of superficial leiomyosarcomas and is less common in higher‐grade tumors. Immunohistochemical studies of deparaffinized tissue may show rhabdomyosarcoma cells to be positive for vimentin, which indicates a mesenchymal phenotype. More differentiated cells may be positive for desmin and myoglobin, which is indicative of muscular differentiation. Wide local excision is considered the treatment of choice with radiation and chemotherapy being offered to patients with positive margins or nodes, or those with bulky disease.^18^, ^19^, ^20^ Radiotherapy can be considered for larger tumors and/or positive margins, if anatomically feasible. Adjuvant chemotherapy has not been proven to be effective and remains investigational. Treatment for metastatic disease is palliative. Active agents include doxorubicin, ifosfamide, gemcitabine, and docetaxel. We recommended adjuvant radiotherapy of tumor bed to our patient hoping for a better prognosis though radio‐sensitivity of leiomyosarcoma is doubtful. Due to rarity of the condition, nothing definite can be told regarding management and prognosis.^4^ Usual treatment followed is nephroureterectomy with a bladder cuff resection as done in ureteral carcinoma. This does not imply that leiomyosarcoma is multicentric or has intraluminal metastasis, but accurate preoperative diagnosis is not possible. Prognosis is also unclear due to paucity of data. Not all ureteral leiomyosarcoma are fast‐growing.

Since there is no mode of definitive treatment for this rare disease, one must extrapolate from modes of treatment of leiomyosarcoma of the ureter and epithelial tumors of the ureter. In our case, surgical excision of the ureteric stricture with safety margin is performed, surrounding tissues and iliac lymph nodes are removed.

## 3 CONCLUSION

Although leiomyosarcoma is rarely seen in urinary tract, it should be considered in the differential diagnosis of ureteral stricture disease and tumors.

## CONFLICT OF INTEREST

The authors declared no potential conflict of interest with respect to the research, authorship, and/or publication of this article.

## AUTHOR CONTRIBUTIONS

HA wrote the manuscript, performed the operation, and managed the patient's perioperative course. HA, YE treated the diabetes insipidus and PK examined the tumor histopatholgy. YE and PK gave the final approval of this manuscript and read and approved the final manuscript.

## ETHICAL APPROVAL

All procedures performed in this study were in accordance with the ethical standards of the Institution and/or National Research Committee and with the 1964 Declaration of Helsinki and its later amendments or comparable ethical standards.

## Content

Written informed consent was obtained from the patient for the publication of this case report and any accompanying images.

## Data Availability

The data that support the findings of this study are available from the corresponding author upon reasonable request.
